# MiRNAs in Lung Cancer: Diagnostic, Prognostic, and Therapeutic Potential

**DOI:** 10.3390/diagnostics12071610

**Published:** 2022-07-01

**Authors:** Javaid Ahmad Wani, Sabhiya Majid, Zuha Imtiyaz, Muneeb U. Rehman, Rana M. Alsaffar, Naveed Nazir Shah, Sultan Alshehri, Mohammed M. Ghoneim, Syed Sarim Imam

**Affiliations:** 1 Department of Biochemistry, Government Medical College (GMC-Srinagar), Karan Nagar, Srinagar 190010, J&K, India; wanijavaidstd@gmail.com; 2 Clinical Drug Development of Herbal Medicine, College of Pharmacy, Taipei Medical University, Taipei 11031, Taiwan; zuhabazaz161991@gmail.com; 3 Department of Clinical Pharmacy, College of Pharmacy, King Saud University, Riyadh 11451, Saudi Arabia; 4 Department of Pharmacology & Toxicology, College of Pharmacy Girls Section, Prince Sattam Bin Abdulaziz University, P.O. Box-173, Al-Kharj 11942, Saudi Arabia; rmalsaffar2020@gmail.com; 5 Department of Pulmonary Medicine, Government Medical College (GMC-Srinagar), Karan Nagar, Srinagar 190010, J&K, India; naveednazirshah@yahoo.com; 6 Department of Pharmaceutics, College of Pharmacy, King Saud University, Riyadh 11451, Saudi Arabia; salshehri1@ksu.edu.sa; 7 Department of Pharmacy Practice, College of Pharmacy, AlMaarefa University, Ad Diriyah, Riyadh 13713, Saudi Arabia; mghoneim@mcst.edu.sa

**Keywords:** miRNA, lung cancer, diagnostics, therapeutics

## Abstract

Lung cancer is the dominant emerging factor in cancer-related mortality around the globe. Therapeutic interventions for lung cancer are not up to par, mainly due to reoccurrence/relapse, chemoresistance, and late diagnosis. People are currently interested in miRNAs, which are small double-stranded (20–24 ribonucleotides) structures that regulate molecular targets (tumor suppressors, oncogenes) involved in tumorigeneses such as cell proliferation, apoptosis, metastasis, and angiogenesis via post-transcriptional regulation of mRNA. Many studies suggest the emerging role of miRNAs in lung cancer diagnostics, prognostics, and therapeutics. Therefore, it is necessary to intensely explore the miRNOME expression of lung tumors and the development of anti-cancer strategies. The current review focuses on the therapeutic, diagnostic, and prognostic potential of numerous miRNAs in lung cancer.

## 1. Introduction

Lung cancer is the second most prevalent cancer with an incidence rate of 11.4% (22,06,771 cases) [1,2] and is a leading cause of cancer-related mortalities worldwide with a rate of 18% (17,96,144 cases) [1,2,3]. The age-adjusted incidence rate per 1000 in south-central Asian countries is 9.4 in males and 3.4 in females [4]. Lung cancer progression is a complex and multistep process that leads to the sequential development of genetic and molecular defects. It begins with 9p and 3p chromosome loss and ends with cyclin D1 and E over-expression [5]. Small cell lung cancer (SCLC) is less prevalent (16.8%) but more lethal than non-small cell lung cancer (NSCLC), which is more prevalent (80.4%) and heterogeneous among the population [6]. Chemotherapy using cytotoxic drugs and radiotherapy is the standard treatment option for lung cancer. Later, precise therapeutic drugs are employed to target specific genetic aberrations in lung tumors, guided by tumor genomic profiling. For instance, epidermal growth factor receptor (EGFR) mutation was the first genetic aberration targeted by tyrosine kinase inhibitors (TKIs), such as erlotinib and gefitinib [7,8]. The other strategy is immunotherapy, which prevents immune system compromise to the tumor (immunological anergy) and stimulates the immune system against the tumor. The immune checkpoint proteins that cause immunological anergy include programmed cell death protein1 (PD1), programmed death ligand1 (PDL1), cytotoxic T lymphocyte-associated protein4 (CTLA-4), etc. For instance, the monoclonal antibody Ipilimumab is used against CTLA-4, enhancing T-cell activation against tumors [9]. 

The current diagnostic methods for lung cancer include radiologic investigations, such as chest X-ray (CXR), low dose computer tomography (LDCT), positron emission tomography and computer tomography (PET-CT), and pathological investigations like lung cancer biopsy, bronchoscopy, etc. [10,11]. However, several drawbacks are associated with existing diagnostic regimens, such as low CXR sensitivity and overdiagnosis of lung cancer associated with LDCT, which results in psychological stress and unnecessary treatments [12]. Due to the physiological variations in (18F)-fluoro-2-deoxy-D-glucose uptake, malignancies that are PET negative, such as SCLC, and carcinoid tumors [13], and non-malignant conditions that respond to PET-positive, like infection, and inflammation [14], confound PET-CT interpretation. Also, technical artefacts and quantitative errors cause misinterpretation of PET-CT results [15,16,17]. Lung bronchoscopy is an invasive diagnostic method, and recently individuals have been predisposed to respiratory infection after undergoing bronchoscopy for lung cancer confirmation [18].

The use of integrative therapeutic strategies for lung cancer, especially immunotherapy and molecular-targeted therapy, has significantly improved the survival of specific populations over the past 20 years. Nevertheless, the efficacy of existing lung cancer therapies is inadequate owing to the lack of effective diagnostic techniques and the development of drug resistance, which, together, restrict the increase in survival after treatment. Thus, there is a need to understand lung cancer biology and explore new effective biomarkers and treatment approaches to lower the burden of lung cancer [19]. MiRNAs are small 20–22 nucleotide single-stranded non-coding RNA molecules, discovered in 1993 [20]. Later, in 2004, Takamizawa et al. highlighted the relationship between miRNA expression and lung cancer [21]. The primary function of miRNA is to control gene expression, mostly at the post-transcriptional stage [22]. In lung cancer cells and tissue, miRNAs exhibit an altered pattern of expression [23,24]. MiRNAs may be pro-oncogenic or anti-oncogenic and can regulate cell invasion, proliferation, migration [25,26], cell viability [25], epithelial-to-mesenchymal transition (EMT) [27], metastasis [28], and therapeutic resistance [29] in lung cancer. For example, miRNAs such as miRNA-195 and miRNA-497 inhibit the progression and formation of colonies in lung cancer by increasing the expression of TGF-β [30]. At the same time, miRNA-196b-59 promotes the progression and formation of colonies in lung cancer by decreasing FAS expression [31]. MiRNA-143-3p promotes metastasis of lung cancer cells to the brain by stimulating N6-methyladenosine [32], while miRNA-192-5p prevents metastasis of lung cancer cells to bones by negatively regulating TRIM44 [33]. 

The currently emerging clinical investigation method for lung cancer is miRNA expression analysis in tumor cells and biofluids like blood, due to its minimally invasive nature and high potential for early lung cancer detection [24]. Since a single miRNA targets multiple gene products or pathways, i.e., showing pleiotropic property, it possesses more therapeutic intervention potential than a drug targeting a single protein-coding gene. It also seems that single target therapeutic intervention is more prone to acquired chemo-resistance [34]. Specific miRNAs, such as miRNA-432-5p, promote resistance against EGFR inhibitors [29], while miRNA-27b can cause lung cancer chemo-resistance to retreat by down-regulating Snail1-mediated suppression of EMT [35]. As a result, miRNAs may serve as potential biomarkers for lung cancer diagnosis, prognosis, and treatment [36,37]. Thus, miRNAs have the potential to be therapeutic targets in the future, and should be explored as such.

## 2. MiRNA Deregulation in Lung Cancer

Fluctuations in miRNA expression are the dominant consequence of miRNA deregulation in cancer [38]. The expression of different miRNAs has been found to be altered in various lung cancer types, such as NSCLC [39,40], lung carcinoids [41,42,43], lung adenocarcinoma, large cell neuroendocrine carcinoma, and squamous cell carcinoma [44] (Table 1).

The miRNA expression within a cell is controlled by genetic and epigenetic mechanisms (DNA methylation) that regulate the transcription of pri-miRNA, its biosynthesis machinery, or specific transcription factors related to its biosynthesis [147]. The transcription factors of miRNA-processing enzymes (DROSHA and DICER) either promote or repress their transcription, affecting miRNAs’ expressions. For example, transcription of the DROSHA gene was either activated by MYC or repressed by ADARB1 transcription factors [148,149]. MYC binding to DROSHA gene promoter accelerated DROSHA transcription, causing elevated miRNA processing in A549 lung cancer cells [148]. TAP63 transcription factor binding to the DICER promoter facilitates its transcription, and dysfunctional TAP63 was found in many cancers [150]. For example, reduced DICER expression was associated with the development of lung cancer [151]. In addition to DROSHA, dysfunctional miRNA silencing complex (miRISC) constituents, like AGO2 and TARBP2, also affect the gene silencing mechanism.

Unfortunately, a reasonable number of miRNA genes (50%) are present at fragile sites prone to deletion, amplification, or translocation in cancer, which becomes an essential factor for dysregulation of target mRNA that can initiate cancer progression as lung cancer [152,153]. Mutations of miRNA binding sites in the 3’UTR of oncogenic mRNAs or single nucleotide polymorphism (SNP) in the seed sequence of mature miRNAs also increased cancer risk, due to decreased target control. For example, genetic tumor profiling revealed that people with KRAS who have a SNP in the 3’ UTR are more likely to develop NSCLC. This may be because KRAS is now not controlled by the miRNA [154]. Hypoxic conditions within cancer cells stimulate EGFR to phosphorylate AGO2 at Tyr393, resulting in decreased AGO2 function that causes decreased DICER–AGO2 interaction, leading to decreased miRNA maturation and function [155]. Epigenetic modifications of chromatin within cancer cells also lead to miRNA dysregulation. MiRNA genes are controlled by epigenetic modulators like DNA methylation and histone modifications [156]. Epigenetic modification, like methylation of the miR-126 promoter sequence, decreases its expression, promoting lung carcinogenesis [157,158]. For example, it is found that promoters of miRNA genes containing CpG islands are heavily methylated in lung tumors, as in the case of miR-34a and miR-34b-miR-34c transcribed from chromosome 1p36 and chromosome 11q23, respectively [159].

Similarly, promoter methylation has been linked with downregulation of miR-200c that stimulates poor differentiation, poor E-cadherin expression, and lymph node metastasis in NSCLC [160]. Davalos et al. (2012) investigated the methylation status of CpG islands present in the regulatory sequences of miR-200c/141 and miR-200b/200a/429 and observed that they are heavily methylated in transformed lung cells, compared to healthy cells, which encourages EMT [161]. 

ZEB1and ZEB2 transcription activators promote gene expression of the mesenchymal phenotype and also discourage known EMT [87]. Histone modifications also have a vital role in miRNA dysregulation via miRNA gene transcription change, and therefore some miRNA genes were downregulated independently of hypermethylation. For example, decreased expression of miR-212 has been frequently linked with H3K9me2 and H3K27me3 histone modification in lung cancer cells [162]. In addition to epigenetic modifications, SNPs in promoter region and RNA editing affect miRNA biogenesis and function in NSCLC [163,164,165]. 

## 3. Tumor Enhancer miRNA

Tumor enhancer miRNA levels are generally elevated in tumors like conventional oncogenes. They are also referred to as oncomiRs, whose deregulation, usually over-expression, triggers carcinogenesis since they negatively regulate tumor suppressor genes, with or without affecting cell differentiation or the apoptosis transcriptome. Most solid tumors experience deregulated miRNA levels [166]. Here we have discussed some well-known oncomiRs (Table 2).

### 3.1. MiR-21

Mature miR-21 is encoded from chromosome 17q23.2, a conserved single gene locus [174,175]. Lung cancer patients demonstrated significantly higher miR-21 expression than normal controls [176]. The possible reasons for frequent over-expression of miRNA-21 force us to think about activating mutations in its regulatory sequence or amplifying its genomic locus. MiR-21 is a crucial protagonist in cell growth, proliferation, migration, invasion, and metastasis. The upregulation of miR-21, commonly in solid tumors [177,178,179], suppresses the expression of tumor suppresser genes like RECK, NFIB, TIMP3, TPM1, STAT3, etc. [60,61,62]. MiR-21 also has diverse targets of negative regulators of the cell growth (Spry1, Spry2, Btg2, and Pdcd4) pathway and apoptosis (Apaf1, Faslg, RhoB, and Pdcd4) pathway [63].

Anti-miR-21 treatment of the A549 lung cancer cell line inhibits proliferation [180]. It may discourage malignant NSCLC spreading to distant sites by releasing the PTEN tumor suppressor gene product, a direct target of miR-21 [181]. Interestingly, miR-21 over-expression was exaggerated in EGFR gene mutant cases and suppressed by treating EGFR tyrosine kinase inhibitors in never-smoking lung cancer patients [182]. The lung adenocarcinoma-derived cell line (pc-9) becomes Gefitinib-resistant when miR-21 is forcedly overexpressed [183], and miRNA upregulation is associated with the overall survival of lung cancer patients [184,185]. Another study found that miR-21 interferes with EMT by negatively regulating the expression of SMAD7, which is a crucial inhibitor of TNFα [186]. MiR-21 deletion/knockout sensitizes cells to DNA-damaging chemotherapy, which suggests inhibition of miR-21 by anti-miRNA agents improves chemotherapeutic action in lung cancer [63]. MiR-21 is an essential anti-apoptotic miRNA, positively regulated by EGFR cell signaling. Interestingly, miRNA controls protein-coding mRNA, and long non-coding RNA (LncRNA), like GAS5 controls the miRNA expression. The in-vitro cell culture and xenograft mouse model study conducted by Ziqiang Zhang et al. observed a double-negative feedback loop in which GAS5 negatively regulates the expression of miR-21 [187]. Further investigation found that forced under-expression of GAS5 increases chemoresistance of the NSCLC cell line (H157, H460) cisplatin (DDP), since GAS5 sponges the miR-21, relieves PTEN mRNA from inhibition, and improves its expression within the cell [188].

Lung cancer patients exhibit increased levels of miRNA-21-5p in their blood circulation [54]. MiRNA-21-5p inhibits the expression of SMAD7, which may enhance lung cancer cell proliferation, migration, and invasion [189]. Inhibiting miRNA-21-5p can effectively prevent the progression of lung cancer [190]. Thus, miR-21 can be utilized as an effective non-invasive biomarker for the diagnosis and prognosis of lung cancer [176].

### 3.2. MiR-17-92

Since the miR-17-92 cluster is found to have a well-documented contribution to the development of malignant diseases and is involved in the development of multiple organs in mammals, it has been extensively studied [191]. The miR-17-92 cluster is found on chromosome 13(q31.3) within the third intron of the C13 or f25 gene [192], which was found to be a first tumor enhancer miRNA [193]. The miR-17-92 cluster is transcribed as a polycistronic precursor transcript containing tandem stem-loop structures trimmed by dicer enzyme to generate mature miR-17-92 family members: miR-17, miR-18a, miR-19a, miR-19b-1, miR-20a and miR-92a [194]. The leading oncogenic player of miR-19-72 was miR-19, which directly targets the PTEN gene, and thus membrane-bound PI 3,4,5 triphosphate is not dephosphorylated, leading to uncontrolled proliferative AKT signaling [195]. About 30 targets of the miR-7-92 cluster were found, especially tumor suppressor genes like PTEN, RB1, P53, IRF2 SPRY4, etc., validated by the luciferase enzyme assay in lung cancer. The over-expression of miR-17-5p and miR-18a are also correlated with shorter survival in lung cancer individuals [102,111,112,113].

Mir-17-5p shows substantial upregulation in patients with NSCLC compared to normal controls, signifying this miRNA’s tremendous clinical importance in NSCLC diagnosis [196]. In lung cancer patients, miR-18a levels were found to be positively correlated with clinicopathological characteristics, such as TNM (lung cancer staging), while being negatively correlated with a therapeutic response (radiotherapy) [197]. A p53-induced lncRNA called TP53TG1 is under-expressed in lung cancer cells and tissues. Further experiments found that it decreased miR-18a expression and increased cisplatin sensitivity in the lung cancer cell line(A549) [198]. MiR-19a is a critical oncogenic family member of the miR-17-92 cluster since it promotes proliferative EGFR signaling in lung cancer [108]. The forced over-expression of miR-19a promotes EMT [199], reduces survival in NSCLC patients [145], and increases gefitinib-resistance in NSCLC cells via directly targeting the c-MET gene, both in in vivo and in vitro experimental systems [109]. MiR-20a and E2F1 form a negative feedback loop augmented by c-MYC [200,201,202]. Qin et al. discovered that miR-20a is over-expressed in malignant lung cells and tissues while RUNX3, a downstream effector of the TNF-α pathway, is downregulated. MiR-20a inhibits TNF-α pathway activation by directly targeting RUNX3 [169]. The introduction of miR-92a into NSCLC cell lines has the same effect as the knockdown of the PTEN tumor suppressor gene. Further research found PTEN and RGS3 are the direct targets of miR-92a and miR-92a-enhanced cell survival, caspase-3 activity, cell proliferation, and metastasis of lung cancer cells [170,203]. Aarati et al. found that over-expressed miR-92a-2 was associated with poor prognosis and resistance to chemotherapy drugs and miR-147 and miR-574-5p [204]. The miR-17-92 complex might be a potential biomarker for diagnosing NSCLC [205].

### 3.3. MiRNA-221

MiR-221/222 is transcribed from the X chromosome (Xp11.3). Its target selectivity remained highly conserved in vertebrates during evolution as it possesses the same seed sequence in humans, mice, and rats [206]. When the April/TRAIL sensitized human lung cancer cell line (H460) is transfected with miR-221/222, it becomes resistant [92]. Culturing TRAIL-resistant cells in the presence of miR-221/222 inhibitors improved TRAIL sensitivity [92]. MiR-221/222 reduces the expression of cyclin-dependent kinase inhibitors, such as p27Kip1. A reduced expression of p27kip1 in NSCLC cells may be a reason for decreased sensitivity towards TRAIL-induced apoptosis. The coincidence of over-expression of miR-221/222 with TRAIL resistance in epithelial cancers [206,207,208] found additional targets of this miRNA, linked to cell proliferation and apoptosis, such as TIMP3 [207], PUMA [93], and PTEN that promotes proliferation in A549 cells [209]. MiR-221 and miR-222 are subjected to activation by MET through the c-JUN transcription factor [130]. Interestingly, miR-130a improves TRAIL sensitivity by downregulating miR-221/222, directly targeting MET gene expression [210]. MiR-221 expression and quantity in patient tissue or serum predict the extent of lymph node metastasis and disease progression in non-small cell lung cancer [211]. However, Sun et al. found that miR-221 negatively regulates the expression of HOTAIR LncRNA and promotes apoptosis of NSCLC cells [212].

MiR-221 expression has been elevated in patients with benign metastasizing leiomyoma (BML) lungs [213]. In NSCLC patients, miR-221 levels increase significantly in plasma, suggesting its potential as a therapeutic target or non-invasive marker for early diagnosis and screening of NSCLC [214]. Wang et al. (2020) also suggested miR-221 as one of the biomarkers for early detection of NSCLC [215].

## 4. Tumor Suppressor miRNA

On analyzing miRNA of malignant cells, some miRNAs are downregulated. These types of miRNAs are considered tumor suppressor genes (Table 3). They discourage the process of cancer progression by specifically targeting oncogenes and, in other cases, genes that play a role in cell differentiation or apoptosis. Several miRNAs are currently considered tumor suppressor genes, for example, miRNA let-7. Here, we discuss a few of them.

### 4.1. MiR-Let-7 Family

Reinhart first discovered the 21 nucleotide non-coding RNA (let 7) when he studied developmental timing in *C. elegans* [228]. Later, it was found that a low level of let-7 correlated with the shorter survival rate of lung cancer patients [21]. It has been found that the Let-7 family constitutes 11 members, among which six members are located in genetically fragile regions of the genome that have a high propensity to genetic alteration [152]. The causes of let-7 downregulation may be a direct epigenetic or genetic aberration in its gene or be due to the activity of repressors of its transcription [229]. Let-7 has a prominent role in diminishing the effects of proliferative, inflammatory, and anti-apoptotic pathways via downregulating their downstream effectors, such as KRAS, c-MYC, CDK6, HOXA9, TGFBR1, BCL-XL, and MAP4K3, thereby creating an anti-malignant environment within the cell [97]. Several Let-7 members are transcribed from genomic regions frequently found missing in lung cancer individuals, such as let-7a, let-7c, and let-7g [230]. MiR-let-7 acts as a tumor suppressor miRNA that downregulates KRAS expression [231].MiR-let-7 mimics injected into the genetically engineered NSCLC mouse model dramatically reduced its tumor area, size, and metastasis compared to the placebo group, suggesting it could be a specific therapeutic tool for lung cancer [232], and is associated with better prognostic value in lung cancer [233]. The transfection of Let-7c and miR-200c in A549 cells improved erlotinib sensitivity, but the same results were obtained when treated with Hh (hedgehog) signaling inhibitor (GDC-0449) [234]. The lowered expression of Let-7 and miR-17 is associated with self-renewal and proliferation by targeting MYC and CDKN1A, which lead to gefitinib-resistance in NSCLC patients [235]. It is found through both in vivo and invitro studies that any imbalance in LIN28 gene expression and Let-7 miRNA expression leads to chemoresistance or radio-resistance in NSCLC, since they interact in a double-negative feedback mechanism [136,236]. 

The Let-7 family shows potential as a non-invasive marker for cancer diagnosis [237]. Lower serum miR-let-7a expression has been strongly associated with poor prognosis and efficacy of radiotherapy in lung cancer brain metastasis [238]. Furthermore, miR-let-7a regulates DICER1 and may be a critical predictive marker of lung cancer brain metastasis [238]. MiR-let-7e is an essential constituent of the lncRNA SNHG4/let-7e/KDM3A/p21 pathway, which has been related to NSCLC development and is possibly one of the vital therapeutic targets for NSCLC [239]. 

### 4.2. MiR-200 Family

Human miRNA-200 is composed of five members, three of them (miR-200a, miR-200b, miR-429) are transcribed from chr12p13 as a polycistronic mRNA, and the rest of them (miR-200c, miR-141) are transcribed from the chr1p36 region [240]. The miR-200 family is commonly recognized as being negative regulators of EMT, since they directly target negative regulators of E-cadherin transcription, such as ZEB1 and ZEB2(zinc finger E-box-binding homeobox) [88,163,221]. Interestingly, miR-200 and ZEB1 form a double-negative feedback loop in which ZEB1 also acts as a transcriptional repressor of the miR-200 family and maintains homeostasis in cell migration, invasion, and EMT [163,241,242]. MiR-200 inhibits angiogenesis, which is required for tumor survival, by lowering vascular endothelial growth factor (VEGF) and vascular endothelial growth factor-receptor1 (VEGF-R1) mRNA levels [243,244]. MiR-200c upregulation may improve radiosensitivity in lung cancer individuals, since it directly targets oxidative stress response genes such as PRDX2, GAPB/Nrf2, and SESN1. This suggests that miR-200c can improve radiotherapy in lung cancer patients [245]. The miR-200/ZEB loop may serve as a prognostic factor for nintedanib sensitivity in malignant lung cells since nintedanib sensitivity is associated with upregulated miR-200a/b, miR-141, and E-cadherin levels and block TGF-β1-induced EMT [246]. Another study found Decitabine discourages TGF-β1-mediated abnormal methylation of the miR-200 gene, which decreases tumor cell migration [247]. A recent study conducted by Kim et al. found that miR-200 directly interacts with QKI (quaking homolog), a kind of STAR protein, and reduces its expression in malignant cells of the lung. MiR-200 and QKI interact in a so-called adverse feedback loop in which QKI knockdown reduces miR-200 expression [248]. 

Recently miR-200 has been reported to exhibit solid diagnostic ability in liquid biopsies of lung carcinoma [249]. The miR-200 family strongly suppresses the metastasis of lung adenocarcinoma. Decreased expression of miR-200 has been observed, especially in mouse lung adenocarcinoma metastasis, which is strongly related to poor survival of the patient. In a mouse model with lung adenocarcinoma (Kras^LSL-G12D/+^; Trp53^flox/flox^), metastasis has been reported drastically promoted by miR-200c/141 deletion, resulting in a desmoplastic tumor stroma that remarkably resembles human metastatic lung carcinoma. Deficiency of miR-200 in lung cancer cells activates neighboring cancer-associated fibroblasts and promotes their proliferation, which increases the capability of cancer cells for metastasis [250].

### 4.3. MiR-206

The genes of human miR-206 (hsa-miR-206) and miR-133 are transcribed from Chr 6p12.2 genomic landmarks adjacent to one another [67]. Hsa-mir-206 is a myomir, which means it is usually expressed in muscles, especially skeletal muscle tissue, and has a prominent role in myogenesis in humans [251]. MiR-206 is found downregulated in individuals with advanced lung cancer. Cell transfection studies have proven that miR-206 has a tumor-suppressive role, i.e., proapoptotic, antimetastatic, and antiangiogenic. MiR-206 directly interacts with the 3UTR of c-MET, EGFR, and Bcl2 in NSCLC cell lines, promoting apoptosis, but discouraging cell proliferation [216,217,218]. MiR-206 prevents the suppression of tumor angiogenesis, both in vivo and in vitro, by inhibiting the 14-3-3z/STAT3/HIF-1α/VEGF signaling pathway [252] and preventing normal fibroblast to cancer-associated fibroblast conversion by downregulating VEGFA expression [253]. MiR-206 restoration improved the cisplatin sensitivity in NSCLC cell lines and discouraged the potential of EMT, invasion, and migration by silencing MET gene expression, and repressing the PI3k/Akt/mTOR signaling pathway [254]. Further research found pentose pathway genes (G6PD, PGD, TKT, GPD2) to be direct targets of miR-206, which is the reason for suppression of the growth of H1437 and A549 cell lines. MiR-206 expression is decreased by over-expressing NRF2, leading to increased pentose phosphate pathway gene activity and redirection of carbon flux towards the pentose phosphate pathway and tricarboxylic acid cycle [255].

Ke-gang Jia et al. found that HK2 is the direct target of miR-206 and prevents explicit cancer cell proliferation by neutralizing the Warburg effect [256]. It is found that an LncRNA, called SNHG14, downregulates miR-206 by directly binding it, and a higher expression of SNHG14 promotes cell proliferation, invasion, and migration in NSCLC. Thus, the expression of SNHG14 indirectly encourages the expression of G6PD [257]. Further research found that miR-206 downregulates COROIC protein, a key player in proliferation, metastasis, and invasion of malignant lung cells (A549) and the A549 xenograft model. The same study also observed restoration of the miR-206 inhibitory effect by CORO1C gene knockdown [258].

Lower miR-206 expression decreased patient survival in NSCLC [259]. The LncRNA/WTI-AS/miR-206/NAMPT cluster, with miR-206 as an important component, could be a new key marker for lung adenocarcinoma diagnosis and prognosis [259]. In EGFR mutant lung cancer cells, miR-206 was shown to reduce HGF-induced gefitinib resistance [260]. MiR-206 affects EVI1 expression and activates the Akt/JNK pathway in SCLC to regulate stem cell proliferation and division [261]. MiR-206 is a metastatic tumor suppressor, and it may be used as a therapeutic target in the clinical treatment of NSCLC [257].

### 4.4. MiR-146 Family

The miR-146 family consists of two miRNA members (miR-146a-5p and miR-146b-5p). MiR-146a is transcribed from a long non-coding RNA gene located at chromosome 5(5q33.3), and miR-146b is transcribed from an intron located at chromosome 10(10q24.32) [262]. MiR-146a regulates various immune responses, such as antiviral, inflammatory, and innate immune responses [263]. The results of many studies suggest miRNA-146a is a potent anti-inflammatory miRNA that directly targets COX-2 [45] and FLAP protein [264]. Knockout of the miR-146a locus in mice makes them hypersensitive to bacterial attack [265]. Cell line-based studies found miR-146a mimic transfection results suggest their interaction with an IRAK1 target either enhanced (regulatory T-cell) or suppressed their expression (Breast cancer cell line) [46]. Many functional polymorphisms in the miR-146a gene have been found in human cancers, increasing the risk of cancer development [156,266,267]. A study conducted on Chinese non-smoker women found that miR-146a rs2910164(CG/GG/CC) polymorphism decreased the risk for lung cancer, since the target binding preference of miR-146a has changed and has now acquired the ability to target the 3UTR of the TRAF6 oncogene [47].

MiR-146a has a well-recognized prognostic value, as its expression has been linked to improved overall survival and response to chemotherapy (EGFR-TKI) and has antiproliferative, antimetastatic, and pro-apoptotic properties in NSCLC [86]. Later, Chaohui Wu et al. found that higher serum miRNA-19b and lower levels of miR-146a are associated with poor overall survival, chemoresistance, and advanced TNM in NSCLC individuals [52]. MiR-146a mimic transfection improved cisplatin sensitivity, promoted apoptosis, and inhibited metastasis in A549/DDP via stimulating the JNK2-p53-Bcl2 axis [268]. Another study found that over-expression of miR-146a, or knockdown of cyclin J (CCNJ), produces the same effect: improved sensitivity to cisplatin mediated through inhibition of cell cycle, cell viability, and motility, and promotion of apoptosis in cisplatin-resistant NSCLC cell lines (A549, SPC-A1). This suggests that miR-146a directly interacts with the 3UTR region and decreases the CCJN mRNA level. The absence of this interaction may be the reason for drug resistance to cisplatin in NSCLC. Thus, treating A549/DPP cells with miR-146a mimic could be a novel strategy for solving the cisplatin resistance in NSCLC patients [269].

In lung cancer cells, miR-146-p regulates the expression of claudin-12, which promotes cell survival, migration, and invasion, inhibiting apoptosis and activating signaling pathways, such as Wnt/β-catenin and PI3K/AKT/MAPK [270]. The expression of miR-146a in lung cancer changes and it may serve as biomarker and therapeutic target [271].

## 5. Therapeutic Potential of miRNAs in Lung Cancer

MiRNAs have multiple mRNA targets, and their functional abnormality may lead to pleiotropic effects and a syndrome of disorders, including cancer. Their clinical use as biomarkers and in diagnostics is rapidly emerging. Compared to small drug molecules and protein-based drugs, they have a broader range of targets and traverse the cell plasma membrane, while monoclonal antibodies-based drugs can only target cell surface receptors and circulating proteins. MiRNAs can fine-tune the expression of virtually any gene and its mRNA transcripts [272].

### 5.1. MiRNAs as Therapeutic Agents 

**Figure 1:**
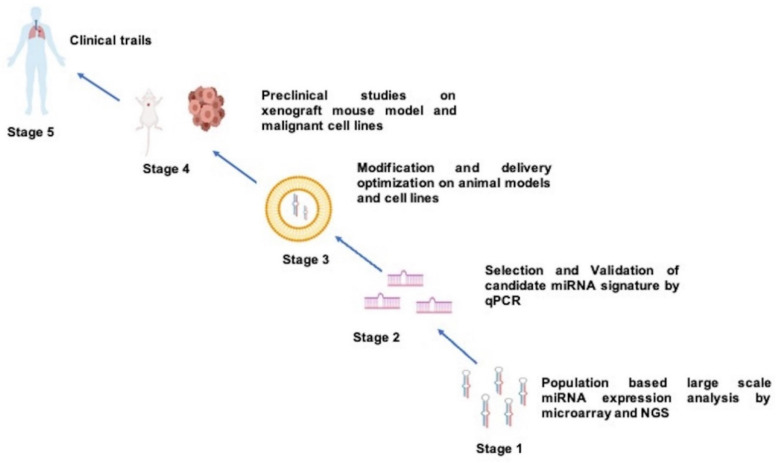
This illustrates different stages of development in miRNA-based drug design for lung cancer.

MiRNAs have a well-recognized role in cancer, and many studies have proved their significance in therapeutics and chemoresistance in cancer. However, the first human trial was conducted on siRNA, which is similar to miRNA, in 2004, and in 2018 the first siRNA drug was approved [273]. The deregulation of miRNAs in cells can be managed by directly introducing miRNAs (restoration strategy) or modulating miRNA expression by therapeutic agents. In the restoration strategy, synthetic dsRNA structures, called miRNA mimics, are used to replace and restore the function of diminished tumor-suppressive miRNA. A synthetic oligonucleotide mimic of miR-34a packaged into liposomal nanoparticles was the first miRNA-based therapy preferentially utilized for cancer [274]. For instance, a combination of Let-7 and miR-34 mimic delivered to a Kras-Trp53 (Kras mutation-p53 deletion) NSCLC mouse model showed promising results, which improved further when complemented with EGFR inhibitor (erlotinib) [67,68]. With the power of genetic engineering and nanotechnology, novel, effective strategies have been developed to deliver miRNA mimics. For instance, a study conducted by Talekar et al. (2016) observed that delivery of wild-type p53 along with miR-125b mimics by dual CD44/EGFR-targeted hyaluronic acid (HA)-based nanoparticles promoted significant macrophage repolarization and stimulated apoptosis in a KP (Kras mutation-p53 deletion) mouse model and SK-LU-1 cells [275]. One study used cationic liposome/CL-pVAX-miR-143 complex (CL-pVAX-miR-143) to deliver miR-143 mimics, which inhibited tumor metastasis in an NSCLC mouse model [276]. Recently miRNA mimics have been delivered by human-derived extracellular vesicles like exosomes not to mouse models but to 3D microfluidic lung cancer models. For instance, an miR-497 mimic was delivered to a 3D lung cancer model [277] (Figure 1). 

### 5.2. MiRNAs as Targets for Therapy

Nucleic acid-based biomedicines, such as oligonucleotides and miRNA sponges, use microRNAs as targets directly or indirectly to achieve therapeutic responses in lung cancer. MiRNA sponges are similar to LncRNA present within the cells, containing multiple binding sites for a specific miRNA. They function by trapping miRNA and preventing binding to its endogenous targets [278]. For instance, long non-coding RNA TUG1 (taurine upregulated gene 1) promotes chemosensitivity of platinum-based chemotherapy by blocking miR-211 function and relieving PTEN from inhibition [279]. Some other examples are anti-miRs antagomirs, LNA (locked nucleic acids), etc. They are designed to block the specific function of miRNA, and their backbone is modified (O2-C4 bridge) or tagged with specific functional groups (2-methoxyethyl) [280]. These modifications make them nuclease-proof and improve thermostability and target specificity [281]. A study conducted by Fu-Gang Dua et al. found that knockdown of miR-421, both in in vivo and in vitro experimental models, by AMO (antisense morpholino) improved paclitaxel sensitivity significantly [282]. The miRNA repression is also achieved by specific chemical agents that target miRNA biogenesis or discourage miRNA-target interaction. Some small inhibitory molecules may target and block miRNA interaction with the RISC complex and possess anti-tumor properties [278]. A cell line-based study conducted by Xigan He et al. found that Docetaxel (a semisynthetic analogue of paclitaxel) discourages proliferation through upregulation of miR-7 in NSCLC cell lines. However, the underlying mechanism is not known [283]. Small molecules also directly target microRNA secondary structure. They directly interact with miRNA precursors to stop Drosha or Dicer cleavage. It is found that aminoglycosides directly interact with RNA secondary structures. Recent findings regarding curcumin found it to have anti-lung cancer properties via downregulation of miR-21 and upregulation of onco-suppressive miR-192-5p and miR-215 [284,285]. Another similar study found that curcumin suppresses the metastasis of NSCLC by stimulating miR-206 expression and discouraging the mTOR signaling pathway [286]. 

## 6. MiRNAs as Potential Lung Cancer Biomarkers

MiRNAs may develop as biomarkers in coming years since they possess remarkable stability in various specimen types [287] and are resilient to extremes of pH and temperature, and they exhibit high specificity, reproducibility, and robustness in expression patterns [288]. MiRNAs may now be added to the panel of possible biomarkers due to their reduced size and the availability of assays that can accurately assess their level, such as qRT-PCR, microarray, and others [289].

### 6.1. MiRNAs as Diagnostic Biomarkers

Lung cancer diagnosis and prognosis can be precisely determined by analyzing several miRNA expressions levels simultaneously (miRNA signatures) [290,291]. Many studies on lung cancer patients unraveled many unique miRNA signatures that are useful for diagnosis, possessing better sensitivity and specificity [292,293,294]. Most studies conducted on serum or plasma found that circulating cell-free miRNA (cfmiRNAs) may be an ideal screening agent for early lung cancer diagnosis [295].

MiRNA expression studies may be exploited to recognize the unknown origin of metastatic tissue. For example, Rosenfeld conducted a miRNA analysis of 22 most common solid tumors and developed a 48-miRNA classifier to identify the origin of unknown primary cancers with 81% sensitivity [296]. MiRNA dysregulation may be perceived at any stage, starting from initiation to progression, allowing us to observe dynamic changes in real-time [297]. These discoveries raise hopes for minimally invasive and early lung cancer diagnosis by exploiting cell-free miRNA expression behavior (cfmiRNA).

Over-expression of miRNA-21 in sputum, a well-recognized EDGF-regulated anti-apoptotic factor, easily distinguished NSCLC individuals from cancer-free individuals [298]. Several unique miRNA signatures, having diagnostic or prognostic importance, were observed in sputum [299]. A metanalysis study conducted by Jipei Liao et al. on miRNA-based lung cancer diagnosis found an integrated panel of biomarkers consisting of both plasma miRNA and sputum miRNA significantly increase the sensitivity and specificity of a lung cancer diagnosis. On further investigation, the performance of the integrated panel of biomarkers was found to be independent of histology and stage of NSCLC, and patients’ age, sex, and ethnicity [300].

Since lung cancer is highly heterogenous and the miRNA species produced by each type are different, these factors can also help classify lung cancer subtypes. MiRNA profile was found to be specific to a histological subtype of cancer, and this property is exploited to discriminate among different histological subtypes of cancer. Many studies on miRNA proved robust in discriminating among lung cancer subtypes [24,301,302]. These miRNA-based clinical studies were of good diagnostic value since squamous cell carcinoma (SqCC) and adenocarcinoma (AD) originating from different cells of lung tissue require different treatment [303]. MiRNAs could also serve as a marker to differentiate primary lung tumors from lung metastases originating from other locations by analyzing the expression of some specific miRNAs. MiR-182 was most significantly over-expressed in primary lung tumors, while miR-126 was over-expressed in lung metastases originating from other tissues of the body [304] (Figure 1).

MiRNAs such as miR-23a and miR-let7i might be clinically valuable biomarkers for the diagnosis of NSCLC [305]. Similarly, miR-21-5p, miR-150, miR-210, and miR-1290 can be utilized as useful early diagnostic and prognostic biomarkers in NSCLC [306]. The panel of miRNAs, including miR-30a-3p, miR-30b-5p, miR-30c-5p, miR-34a-5p, and miR-4286a, might serve as novel biomarkers for the diagnosis and prognosis of lung cancer [307]. One more recent study reported 5 miRNA-based panels (hsa-miR-31, hsa-miR-34c, hsa-miR-196b, hsa-miR-653, and hsa-miR-891a) as potential biomarkers for the diagnosis and prognosis of lung cancer [308]. Likewise, Yu-Long Zhao et al. (2022) reported serum miR-205-5p as a new and useful diagnostic biomarker for lung cancer [167]. ]. MiR-3182 can be utilized as a potential diagnostic biomarker for lung cancer [309]. 

**Figure 2:**
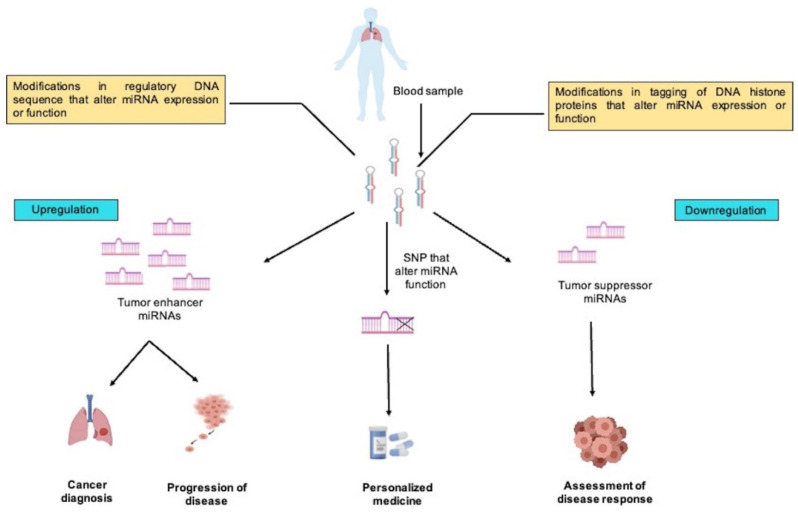
This figure illustrates tumor suppressor miRNAs (targeting oncogenes), tumor enhancer miRNAs (targeting tumor suppressors), and SNP that affect miRNA function (SNPs that render tumor suppressor miRNAs functionless) in the clinical significance of miRNAs in lung cancer. MiRNAs can be used in differential diagnosis of lung cancer subtypes, predicting chemoresistance, radio resistance, survival rate, patient response to treatment, and targeted medicines given to targeted persons.

MiRNA signature utilization for cancer screening has reached the pre-clinical and clinical testing stages. The mir-Test is a pre-clinical effort utilizing miRNA for cancer diagnosis [310]. This test aims to formulate a sensitive, non-invasive method of detecting early lung cancer in a high-risk group (heavy smokers greater than 50 years of age). To establish ideal miRNA biomarkers for clinical use, such as early lung cancer diagnosis, a heavy bulk of miRNA-associated data, generated from miRNA studies conducted on a much larger sample size and a standard operating protocol with standardized platforms and data analysis methods, is essential. Markers established under such conditions may have a higher success rate for clinical adaptation (Figure 2).

#### Exosomal miRNAs as Diagnostic Biomarkers

Pan and Johnstone first discovered exosomes in 1983 during their sheep reticulocyte-related experiment [311]. Exosomes are the smallest vesicles among the extracellular vesicle group, whose diameter may range from 0.03 µm to 0.1 µm [312]. Exosomes act as cargo, transferring their biologically active components [313], such as miRNAs, mRNA, DNA fragments, etc. into targeted cells, playing the role of a cell-to-cell communicator, [314,315,316] like tumorigenesis, carcinogenesis, metastasis, and drug resistance, in cancer. Exosomes form a large proportion of circulating vesicles, from which cancer-associated exosomal miRNAs are purified with high sensitivity and specificity [317]. Tumor-derived exosomal miRNAs are novel diagnostic and predictive biomarkers for overall survival, cancer relapse, and drug resistance. Exosomal miRNAs are considered better biomarkers than non-exosomal miRNAs. Exosomes released by cancer cells harbor cancer-specific miRNAs, RNA, DNA fragments, and membrane proteins, promoting their cancer specificity [318]. Besides, exosomal miRNAs have excellent stability and anti-degradation ability [39].

Exosomal miRNAs are better diagnostic and prognostic biomarkers in lung cancer than non-exosomal circulating miRNAs. Exosomal miRNAs have been found significantly elevated in lung carcinoma patients compared to benign pulmonary diseases. For instance, the circulating blood exosomal miRNAs (miRNA-361-3p, miRNA-625) help in discriminating malignant lung lesions from benign lung lesions [79]. Another study for diagnosing and screening lung cancer found serum exosomal miRNAs (miRNA-200b-5p, miRNA-378, miRNA-502-5p, miRNA-629, miRNA-17, and miRNA-100) were significantly lower in pulmonary granuloma and healthy smokers compared to lung adenocarcinoma subjects [319]. Apart from this, a study by Munagala et al. found serum exosomal miRNAs for predicting lung cancer recurrence or relapse. Based on in vitro cell culture and animal models, 77 exosomal miRNAs were found dysregulated. Of these, 47 were upregulated, and 30 were downregulated. Mirna-21 and miRNA-155 showed significant upregulation in recurrent tumors compared to primary tumors [320]. Peripheral blood exosomes were shown to have 30 specific molecular markers. Thus, exosomes and associated molecules may provide a theoretical basis for determining biomarkers for diagnosing lung cancer at an early stage.

## 7. MiRNAs as Lung Cancer Prognostic Biomarkers

MiRNA analysis of tumors by microarray and qRT-PCR has been found helpful in predicting clinical outcomes, such as response to cancer treatment, cancer relapse, and overall survival [289]. ] Takamizawa et al. discovered for the first time that a reduced level of Let-7 is significantly correlated with worsened prognosis after curative resection in lung cancer subjects [21].

### 7.1. MiRNAs as Biomarkers of Survival

A meta-analysis by S. R. Lamichhane found that miR-21, miR- 155, miR-148a, miR-148b, and miR-let-7 are consistently up or downregulated in NSCLC and show significant prognostic potential in the diagnosis, treatment, and follow-up of NSCLC [321]. Another meta-analysis by Wendi Xiao et al. found downregulation of miRNA-26b, miRNA-381, miRNA-146α, miRNA-148α, miRNA-204, miRNA-374α, miRNA-638 and miRNA-148b) and upregulation of miRNA-125b, miRNA-21, miRNA-141, miRNA-200c, miRNA-197, miRNA-41, miRNA-370, miRNA-376α, miRNA-192 and miRNA-662 are consistently linked to poorer overall survival of lung cancer patients [322]. Further investigation revealed their significant correlation with the overall survival of patients [323]. A similar study on SCLC individuals found a triplet miRNA signature (miR-194, miR-608, and miR-9) expression profile in serum that can easily predict overall survival [324].

### 7.2. MiRNAs as Biomarkers of Response to Treatment

Circulating miRNAs, including miR-21, mir-126, and miR-513a, may act as predictive markers for platinum therapy response in NSCLC besides having potential for diagnosis [325]. Zhu et al. (2022) reported a higher expression of miR-1274a associated with poor prognosis, which might be utilized as a potential prognostic biomarker in NSCLC [326]. We can predict which individuals would respond better to targeted therapy or chemotherapy, and develop resistance to treatment, and so modify therapeutic strategy accordingly based on miRNA expression analysis. Resistance of cancers to radiotherapy treatment can also be predicted based on miRNA expression in cancer. When Ma et al. treated four malignant lung cells with increased doses of radiation, they found a 2.5-fold upregulation of miR-95 in the most radioresistant cell line [327]. A study conducted on advanced NSCLC individuals found a specific group of patients that responded well to high-dose radiation therapy. On further investigation, this group possesses a unique miRNA expression pattern compared to poor respondents [328] (Figure 2)

## 8. Challenges in Use of miRNAs as Theragnostic Agents

Many pre-analytical and analytical variables significantly affect the diagnostic and prognostic potential of miRNA expression in lung cancer. Some of them are inherent to miRNAs, such as the addition or deletion of nucleotides, SNPs, isomers, and GC content of miRNAs, which affect miRNA recovery, and change miRNA sequence, and, thus, significantly affect miRNA profiling. Other factors are method used for isolation, storage conditions, and type of profiling method used. It should be noted that cell-free miRNAs (cf-miRNAs) have a non-homogenous origin, i.e., released miRNAs originate from residing cells and endothelial cells. This effect masks the number and level of miRNAs liberated by tumor-derived cells in biofluids. Also, visceral organs (lungs, liver, and kidney) that experience an elevated blood hydrostatic pressure may be a possible source of liberation [329,330,331]. The heterogeneity of miRNA expression is another hurdle. For instance, both inflammation, which is a well-known cancer hallmark, and hypoxia are frequent conditions in the tumor microenvironment, significantly perturbing miRNA expression and creating a foggy picture of candidate miRNAs dysregulated within the tumor [332,333,334,335]. 

Many issues linked with miRNA therapeutics prevent their journey from bench to bedside. Significant issues are associated with specific delivery and poor bioavailability at the target site. Due to their polyanionic nature, transport across the lipid bilayer becomes difficult. Also, vascular barricades, such as tight junctions between the cells, significantly prevent paravascular transport. Their polyanionic nature and conjugation with high molecular weight carriers, like antibodies, for target-specific delivery mean they cannot traverse lipid bilayers spontaneously and remain trapped in lysosomal compartments or are retro-gated back to the plasma membrane [336]. This decreases their bioavailability, and a small fraction remain available for miRISC to act on their target mRNAs in the cytosol. To promote RNA interference (RNAi) machinery activation, the so-called “endosomal escape” of payload (miRNA-conjugate or package) is crucial. Several endos-osmotic agents have been exploited to deliver siRNA and ASO conjugates effectively. Small fusogenic peptides are developed, which can disrupt the endosomal membrane and help release the payload. This concept came after profoundly understanding the mechanisms behind the lysosome escape of pathogens, such as bacteria, viruses, etc. For instance, an HA2 domain of hemagglutinin developed from the influenza virus can be conjugated to promote effective siRNA delivery [337]. Endosomatic polymers are also exploited to promote the “endosomal escape” of siRNA or ASO complexes. In an acidic environment, the hydrophilic polymer is transformed into a hydrophobic polymer causing endosomal membrane partitioning and destabilization [338]. Apart from poor bioavailability, miRNA therapeutics are associated with a high probability of off-target effects, which may compromise target specificity and therapeutic effect and sometimes cause cell death. The frequent off-target effect of therapeutic miRNA is a tendency tobehave as siRNA miRNA, since the seed sequence tolerates many mismatches and decreases the abundance of several non-target mRNAs while still retaining the on-target effect. Another off-target effect is the competitive behavior of therapeutic siRNA with endogenous miRNAs for landing on RNAi machinery; this disturbs the natural equilibrium between RISC and endogenous miRNAs and may lead to unpredictable off-target effects [339]. 

## 9. Conclusions

The study of miRNAs is still in its initial stages. Many issues prevent its transition from batch to bedside, i.e., from research batch to clinics, such as suitable delivery methods, and insufficient understanding of their off-target effects on the human body. There is a need for a better and deeper understanding of targets and biological pathways that affect miRNAs to avoid any possible off-target effects. A large-scale population-based study should be conducted to identify those miRNAs which possess diagnostic, therapeutic, and prognostic potential in lung cancer. Also, there are many such studies whose results contradict each other. People use different specimens, sample collection methods, sample storage conditions, and expression analysis techniques that have a tremendous effect on reproducibility and accuracy and thus the study’s final results. There is a need to develop a standard protocol.

## Figures and Tables

**Table 1:** This table lists some of the miRNAs that have been frequently dysregulated in human lung cancer subjects and were further analyzed for their diagnostic and prognostic potential. The direct targets of these miRNAs are identified experimentally in NSCLC cell lines and animal models. (↑) indicates upregulation while as (↓) indicates downregulation. The number of arrows indicates number of studies in the respective human specimen.

miRNA	Relative Expression Level in Lung Cancer	Clinical Association	Experimental Models	Experimentally Validated Targets	Effect on Lung Carcinogenesis	References
**miR-146a**	Serum (↓↓), serum (↑↑), tissue (↓)	Dual	Xenograft mouse models, cell lines	COX-2, CCJN, FLAP, IRAK1, TRAF6	Suppression	[45,46,47,48,49,50,51,52]
**miR-21**	Serum (↑↑↑↑) tissue (↑↑↑)	chemoresistance and poor prognosis	Xenograft mouse models, cell lines,	RECK, NFIB, TIMP3, TPM1, STAT3, Spry1, Spry2, Btg2, and Pdcd4	Enhancement	[53,54,55,56,57,58,59,60,61,62,63]
**miR-34a/b/c**	Tissue (↓), whole blood (↑)	poor prognosis and relapse	Syngenic mouse model, transgenic mouse model, murine cell lines	Cdh2, Kras, Fn1 SNAIL,	Suppression	[64,65,66,67,68]
**miR-365**	Serum (↓↓)	poor prognosis	Knock out, malignant cell lines	CDC25, NKX2-1, TRIM25	Suppression	[69,70,71,72]
**miR-486-5p**	Serum (↓), tissue (↓↓), endobronchial mucosa (↓),	poor overall survival and chemoresistance	Xenograft mouse model, Knock out, cell lines	ARHGAP5 (RhoA GTPase), mTOR, Pten	Dual	[73,74,75,76]
**miR-361**	tissue (↓), serum (↓	poor prognosis and clinical outcome	cell lines, xenograft mouse model	SH2B1, FOXM1	Suppression	[77,78,79,80]
**miR-615-3p**	Tissue (↓), tissue (↑↑),	Differential diagnosis	cell lines, xenograft mouse model	IGF2	Suppression	[81,82,83]
**miR-200 family**	Tissue (↑, ↑)	Dual	Xenograft mouse model, Knock out, cell lines	ZEB1, ZEB2, VEGF, VEGFR1 PRDX2, GAPB/Nrf2, and SESN1,	Suppression	[84,85,86,87,88]
**miR-221**	Tissue (↑↑), serum (↑↑), serum (↓), plasma (↑↑)	Diagnosis, poor prognosis, and relapse	Xenograft mouse model, Knock out, cell lines	P27^kip1^, TIMP3, PUMA, PTEN, MDM2,	Enhancement	[89,90,91,92,93]
**Let-7a/b**	tissue(↓↓), FFPE tissue (↓↓)	Poor survival rate and clinical outcome	Transgenic mouse, Knock out, malignant cell lines	KRAS, c-MYC, CDK6, HOXA9, TGFBR1, BCL-XL, MAP4K3	Suppression	[21,94,95,96,97,98]
**Let-7e**	Tissue (↓), FFPE tissue (↓↓↓)	Poor survival rate and clinical outcome	Transgenic mouse, malignant cell lines	SUV39H2	Suppression	[21,99,100,101]
**miR-17-5p**	Tissue (↓), tissue (↑), serum (↑), plasma (↓)	Dual	Transgenic mouse	BECN1, TBC1D2	Enhancement	[102,103,104,105]
**miR-19a/b**	Serum (↑↑), tissue (↑)	Poor prognosis	cell lines, xenograft mouse model	c-MET, PP2A, BIM, E-cadherin, ZO-1, *α*-catenin, TNF-α	Enhancement	[90,106,107,108,109]
**miR-18a**	Plasma (↑↑),	Poor prognosis and radio resistance	cell lines	IRF2, ATM, HIF1-α	Enhancement	[102,110,111,112]
**miR-661**	Tissue (↑↑), Serum (↑)	Differential diagnosis and poor prognosis	cell lines	SOX7, RB1, RUNX3	Enhancement	[113,114,115,116]
**miR-26a-5p**	FFPE tissue (↑)	Differential diagnosis	Cell lines,	Integrin-β8, FAF1	Enhancement	[117,118]
**miR-128-3p**	Tissue (↑), tissue (↓)	Differential diagnosis,	Cell lines, xenograft mouse	SMURF2, cpp1, AXIN1, W1F1, SRFP2, DROSHA, DICER	Enhancement	[76,119]
**miR-378**	Tissue (↑↑)	Diagnosis and chemoresistance	Cell lines, xenograft mouse	RBX1, FOXG1, ***RBX1***, ***clustin***	Enhancement	[120,121,122,123]
**miR-93**	Tissue (↑↑↑) Serum (↑)	Diagnosis, Poor overall survival	Cell lines, xenograft mouse	LKB1, TBP2, DAB2	Enhancement	[124,125,126,127]
**miR-135b**	Serum (not significant), tissue (↑)	Diagnosis, EGFR mutations, invasion of visceral pleura	Cell lines, xenograft mouse	LZTS1, LATS1, MOB-1A, Dbf2, βTrCP	Enhancement	[128,129,130]
**miR-16**	Plasma (↑↑) Tissue (↓)	Lung cancer subtype diagnosis, poor prognosis	Cell lines, xenograft mouse, transgenic mice	**TWIST1**, MEK1, HDGF, VEGF,	Suppression	[131,132,133,134,135,136]
**miR-15a**	Serum (↓), Tissue (↓↓↓)	Diagnosis, poor clinical outcome	Cell lines, xenograft mouse	ACSS2, PDL1, FGFR1, DDX3X, SLC1A5 Smad3, FXR1, **BCL2L2**	Suppression	[137,138,139,140]
**miR-7**	Tissue (↓)	Shorter survival and chemoresistance	SCLC cell lines	KIR2.1, ABCC1, PARP1	Suppression	[141,142,143]
**miR-22**	Serum (↑), tissue (↓↓	Differential diagnosis,	cell lines, Murine xenograft mouse	MET-STAT3, ErbB3	Suppression	[144,145,146]

**Table 2:** This table lists some miRNAs, their experimentally validated targets, and their clinical significance in lung cancer. MiRNAs upregulating (↑) in lung carcinoma (tumor enhancer miRNAs) regulate tumor suppressor genes.

miRNAs Expression (Lung Carcinoma)	Effect on Lung Carcinoma	Type of miRNA	Experimentally Validated Targets	Clinical Significance	References
**miR-21** **↑**	Enhancement	Tumor enhancer	RECK, NFIB, TIMP3, TPM1, STAT3, Spry1, Spry2, Btg2, and Pdcd4	Promotes cell proliferation, metastasis and discourages apoptosis	[60,61,62,63]
**miR-205-5p**	Enhancement	Tumor enhancer	TP53INP1	Promotes proliferation and metastasis of lung cancer cells	[167]
**miR-9-5p**	Enhancement	Tumor enhancer	STARD13	Promotes the progression of lung adenocarcinoma cell malignancy	[168]
**miR-221** **↑**	Enhancement	Tumor enhancer	P27^kip1^, TIMP3, PUMA, PTEN,	Promotes TRAIL resistance	[92,93]
**miR-17-92** **↑**	Enhancement	Tumor enhancer	PTEN, RB1, P53, IRF2 SPRY4	Promotes proliferation and metastasis and linked with short survival	[101,102,112]
**miR-95** **↑**	Enhancement	Tumor enhancer	Caspase-3, Caspase-9, Bcl-2,	Sensitises tumor tissue to radiotherapy, enhances apoptosis, and decreases proliferation	[169,170]
**miR-19a** **↑**	Enhancement	Tumor enhancer	c-MET, PP2A, BIM, E-cadherin, ZO-1, and *α*-catenin	Promotes gefitinib-resistance in NSCLC cells and is associated with poor prognosis in NSCLC patients	[107,108,109]
**miR-18a** **↑**	Enhancement	Tumor enhancer	IRF2	Associated with shorter survival and poor therapeutic response	[111,112,113]
**miR-150** **↑**	Enhancement	Tumor enhancer	FOXO4	Associated with metastatic malignant lung cells and tissues	[171]
**miR-619-5p**	Enhancement	Tumor enhancer	RCAN1.4	Promotes tumor angiogenesis and metastasis	[172]
**miR-135b** **↑**	Enhancement	Tumor enhancer	LZTS1, LATS1, MOB-1A, Dbf2, βTrCP	The combined expression of LZTS1, TAZ, and miR-135b predict the prognosis of NSCLC patients.	[171]
**miRNA-182**	Enhancement	Tumor enhancer	FOXO3	Promotes tumor proliferation, chemo- and radioresistance	[173]

**Table 3:** This table lists some miRNAs, their experimentally validated targets, and their clinical significance in lung cancer. MiRNAs downregulating (↓) in lung carcinoma (tumor suppressor miRNAs) regulate oncogenes.

miRNAs Expression (Lung Carcinoma)	Effect on Lung Carcinoma	Type of miRNA	Experimentally Validated Targets	Clinical Significance	References
**miR-146** **↓**	Suppression	Tumor suppressor	COX-2, CCJN, FLAP, IRAK1, TRAF6	Discourages inflammation, associated with better overall survival, better response to chemotherapy (EGFR-TKI)	[45,46,47]
**miR-206** **↓**	Suppression	Tumor suppressor	c-MET, EGFR, Bcl2, VEGFA, VEGF	Discourages proliferation, tumour angiogenesis and promotes apoptosis	[216,217,218]
**miR-34a** **↓**	Suppression	Tumor suppressor	p21 ^WAF1/CIP1^, MDM2,	Adjunctive treatment of NSCLC patients with erlotinib along with miR-34a and Let7b sensitizes its action	[67,68]
**miR-32-5p**	Suppression	Tumor suppressor	SMAD family 3	Inhibits EMT and metastasis in lung adenocarcinoma	[219]
**miR-377**	Suppression	Tumor suppressor	ErbB	Reduces proliferation and induces apoptosis	[52]
**miR-205-5p**	Enhancement	Tumor enhancer	TP53INP1	Promotes proliferation and metastasis of lung cancer cells	[167]
**miR-571**	Suppression	Tumor suppressor	EGFR, MAPK1, PAK2	Inhibits proliferation and induces apoptosis in lung cancer cells	[220]
**miR-486-5p** **↓**	Suppression	Tumor suppressor	ARHGAP5 (RhoA GTPase)	Inversely associated with lymph node metastasis	[171]
**miR-200** **↓**	Suppression	Tumor suppressor	ZEB1, ZEB2, VEGF, VEGFR1 PRDX2, GAPB/Nrf2, and SESN1	Suppresses angiogenesis, EMT and promotes radiosensitivity	[87,88,221]
**Let-7** **↓**	Suppression	Tumor suppressor	KRAS, c-MYC, CDK6, HOXA9, TGFBR1, BCL-XL, MAP4K3	Associated with poor postoperative survival, chemoresistance or radio-resistance	[21,97,98]
**miR-199a-5p**	Suppression	Tumor suppressor	AKAP1	Inhibits NSCLC proliferation and tumorigenecity	[222]
**miR-582**	Suppression	Tumor suppressor	Hippo-YAP/TAZ	Increases YAP/TAZ phosphorylation with a simultaneous reduction in cellular proliferation and promotion of apoptosis	[223]
**miR-582-5p**	Suppression	Tumor suppressor	NOTCH1	Suppresses tumor growth and invasion	[224]
**miR-320a**	Suppression	Tumor suppressor	AKT3	Lower levels correlated with poor prognosis and rate of survival	[225]
**miR-584**	Suppressor	Tumor suppressor	YKT6	Suppresses migration and invasion in NSCLC	[226]
**miR-613**	Suppressor	Tumor suppressor	GJA1	Inhibits lung cancer cell proliferation, migration, and formation of a colony	[227]
